# Ochratoxin A in Moroccan Foods: Occurrence and Legislation

**DOI:** 10.3390/toxins2051121

**Published:** 2010-05-14

**Authors:** Abdellah Zinedine

**Affiliations:** Laboratory of Food Toxicology, National Institute of Health (INH), 27 Avenue Ibn Battouta, P.O. Box 769, Rabat-Agdal, Morocco; Email: zinedineab@yahoo.fr; Email: zinedineab@yahoo.fr; Tel.: +212537771902; Fax: +212537772067

**Keywords:** ochratoxin A, occurrence, legislation, food, Morocco

## Abstract

Ochratoxin A (OTA) is secondary metabolite naturally produced in food and feed by toxigenic fungi, especially some *Aspergillus* species and *Penicillium verucosum*. OTA is one of the most studied mycotoxins and is of great interest due to its toxic effects on human and animals. OTA is produced in different food and feed matrices and contaminates a large range of base foods including cereals and derivatives, spices, dried fruits, wine and coffee, *etc*. Morocco, a North African country, has a climate characterized by high humidity and temperature, which probably favors the growth of molds. This contribution gives an overview of principal investigations about the presence of OTA in foods available in Morocco. Due to its toxicity, OTA presence is increasingly regulated worldwide, especially in countries of the European Union. However, up until now, no regulation limits were in force in Morocco, probably due to the ignorance of the health and economic problems resulting from OTA contamination. Finally, recommendations and future research directions are given required to assess the situation completely.

## 1. Introduction

Mycotoxins are secondary metabolites principally produced by molds of genera *Aspergillus*, *Penicillium *and *Fusarium.* Nowadays, more than 300 mycotoxins are known and their number is constantly increasing, as well as the legislative provisions taken to control their presence in food and feed [1,2]. The most known and studied mycotoxins are aflatoxins (AF), ochratoxin A and *Fusarium* toxins.

Ochratoxin A (OTA) is one the most studied mycotoxins because of the wide range of foodstuffs it contaminates, and also because its occurrence has been reported in foodstuffs all around the world. In North African countries, the foods most suspected to be susceptible to OTA contamination are domestic and imported cereals such as wheat and sorghum, olives, poultry products, and spices [3]. Published data suggest an association between elevated exposure to OTA and cases of human nephropathies in Tunisia and Egypt [4,5,6].

Morocco, a North African country, surrounded by the Mediterranean Sea and Atlantic Ocean, is characterized by a hot and humid climate, which probably favors growth of molds. OTA, a nephrotoxic mycotoxin, usually enters the body via ingestion of contaminated foods. Considering its chemical stability, OTA is of a potential risk for human health. The presence of OTA in foodstuffs results in deterioration of the marketable quality and is responsible for economic losses. Little investigations are available in Morocco on the contamination of foodstuffs by toxigenic fungi; however, the presence of OTA in commercialized foodstuffs was reported [7]. The majority of the total Moroccan population lives on the coasts that are about 4,500 km, and about two million people suffer from chronic diseases of the kidney including chronic renal insufficiency and chronic interstitial nephropathy, especially young people of both sexes. However, the etiology of the diseases is not well established. The prevalence of these diseases is constantly increasing, but the implication of OTA is not yet demonstrated. A preliminary survey reported that the Moroccan population is exposed to OTA [8]. Indeed, 60% of the Moroccan human plasma sampled was positive for OTA (61.5% in the male and 56% in the female population) with an average concentration of 0.29 ng/mL (0.31 ng/mL in males and 0.26 ng/mL in females). 

The aim of this contribution is to give a general review of the principal researches carried out on the occurrence of OTA in food available in Morocco. The regulation of OTA in foods by the Moroccan authorities is also discussed. Finally, research on OTA in Morocco should focus on devising a national program on OTA surveillance and its prevalence in biological fluids of the population to assess the situation completely in the country.

## 2. Toxicity of OTA

OTA is receiving increasing attention due to its toxic effects on humans and animals. Indeed, OTA has been shown to be nephrotoxic, carcinogenic, immunotoxic, genotoxic and teratogenic to all animal species tested. The genotoxicity of OTA has been postulated *in vivo* and *in vitro* [1]. Genotoxic effects such as DNA strand breaks, sister chromatid exchanges, chromosomal aberrations and induction of micronuclei have been observed in mammalian cell systems in response to OTA exposure [9]. The presence of OTA in blood from healthy humans confirms a continuous and widespread exposure. A positive correlation among human nephropathies and dietary OTA exposure or plasma concentrations arises from several epidemiological studies [1,10]. In some Eastern European countries (Bulgaria, Romania, Serbia, Croatia, Bosnia and Hertzegovinia and Slovenia), OTA has been implicated in a human kidney disease, referred to as Balkan endemic nephropathy, characterized by tubule interstitial nephritis and associated with high incidence of kidney, pelvis, ureter and urinary bladder tumors [10]. Consumption of food contaminated with OTA during pregnancy and/or childhood is suspected to induce lesions in testicular DNA that could promote testicular cancer [11]. The mechanisms by which OTA is genotoxic have been recently reviewed [12,13]. One covalent DNA adduct has been identified *in vivo* by ms/ms [14]. OTA was classified as a possible human carcinogen (group 2B) by the International Agency for Research on Cancer since experimental studies demonstrated the evidence for OTA carcinogenicity in animal [15]. A provisional tolerable weekly intake (PTWI) of OTA at 100 ng/kg body weight (b.w.) corresponding to approximately 14 ng/kg b.w./day was established by the Joint Committee FAO/WHO of Experts on Food Additives (JECFA) [16]. Nevertheless, the Panel on Contaminants in the Food Chain (CONTAM) of the European Food Safety Authority (EFSA) recently derived a Tolerable Weekly Intake (TWI) of 120 ng/kg b.w for OTA, which corresponds to a Tolerable Daily Intake (TDI) of 17.1 ng/kg b.w. [17]. However, the Virtually Safe Dose (VSD) of 1.8 ng/kg bw/day proposed by Kuiper-Goodman and Scott [18] that considers tumor formation by OTA as an endpoint would be a more prudent safety level to set for OTA intake.

## 3. Production of OTA

**Figure 1:**
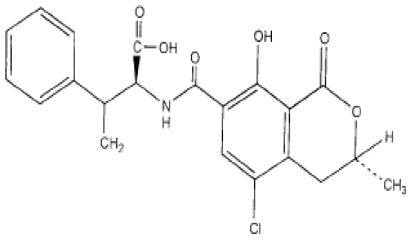
Structure of OTA.

Ochratoxin A is a mycotoxin that was firstly isolated in 1965 by van der Merve *et al.* [19] from maize based products contaminated with *Aspergillus ochraceus*. OTA is chemically known as *N*-{[(3*R*)-5-chloro-8-hydroxy-3-methyl-1-oxo-7-isochromanyl]-carbonyl}-3-phenyl-L-alanine (Figure 1). Four years later, OTA was isolated by van Walbeek *et al.* [20] from the culture of *Penicillium verrucosum*. OTA was described as one of the first groups of fungal metabolites that are toxic to animals, which, with the AFs, launched the distinctive and diverse science of mycotoxicology in the 1960s. Nowadays, two groups of fungi are mainly involved in OTA production. In tropical regions *A. ochraceus* is probably the main source, though several other aspergilli are also able to produce OTA including strains of *Aspergillus alliaceus*, *A. ostianus*, *A. sclerotiorum*, *A. sulphureus*, *A. melleus*, *A. petrakii*, *A. glaucus*, *A. niger*, *A. awamori*, *A. foetidus*, *A. carbonarius*, *A. albertensis*, *A. auricomus *and *A. wentii* [21]. In cool temperate latitudes *P. verrucosum* is responsible, and probably most forms of the fungus can be toxinogenic. Toxigenic species were found to colonize several agricultural products and to be responsible for OTA contamination. Indeed, OTA has been widely detected in cereals including, barley, wheat, maize and oat [22,23], green coffee [24], grape juice [25], and wine [26,27]. OTA contamination of dried fruits was found to be due to the action of black *Aspergilli* in Europe including Spain [28], France [29], the Czech Republic [30] and in other parts of the world such as Argentina [31] and Australia [32]. 

## 4. Occurrence of OTA in Foods Available in Morocco

Cereals represent a staple food for the Moroccan population, therefore bearing high social, economic and nutritional relevance. On average, Morocco consumes six million tons of cereals each year. Moreover, cereals contribute to approximately 12% of the agricultural output and Moroccan households spend 25% of their food expenditure on this kind of product. In addition, by 2020 the Moroccan population will require 8.5 million tons of cereals for the national consumption. Due to drought the country has endured during the last two decades, cereal yield production has been dramatically reduced in the range of 25-85% [33], leading to extensive importation from other countries. Thus, Morocco imports cereals from various countries, particularly from France, USA, Canada, Brazil, Argentina, Russia and Australia. It was reported that approximately 25% of cereals produced in the world are contaminated by mycotoxins [34]. For these reasons, more importance has been given to investigations of the presence of OTA in cereals and derivatives (bread, breakfast cereals *etc*.) from Morocco. However, others foods that are also of importance were investigated by Moroccan scientists especially olives, beverages, dried fruits *etc*. Data about the presence of OTA in foods commercialized in Morocco are presented in Table 1.

**Table 1:** The occurrence of OTA in foods available in Morocco.

Commodities	N	%Positive samples	Range (ng/g or µg/L)	% of samples > MRLs *	Ref.
**Cereals and derivatives**					
*Wheat grain*	20	40	0.04-1.73	-	[36]
	17	11.7	Up to 30.6	2	[37]
*Barley*	20	55	0.04-0.80	-	[36]
*Corn*	20	40	0.05-7.22	5	[36]
*Bread*	100	48	0.14-149	26	[42]
*Rice*	20	90	0.02-32.4	15	[38]
	100	26	0.08-47	14	[52]
*Breakfast cereals*	48	8.3	5.1-224.6	5.8	[41]
**Dried fruits/nuts**					
*Raisins*	20	35	0.05-4.95	-	
*Pistachio*	20	-	-	-	[38]
*Figs*	20	65	0.03-1.42	-	
*Peanut*	20	25	0.10-2.36	-	
*Walnuts*	20	35	0.04-0.23	-	
**Black olives**	25	36	0.62-4.8	-	[49]
	10	100	Up to 1.02	-	[47]
**Beverages**					
*Wine*	30	100	0.028-3.24	3	[43]
*Beer*	5	-	-	-	
*Fruits juices*	14	7.1	1.16	-	

* MRLs: Maximum Residues Limits fixed by the European Union.

### 4.1. Raw cereals

By using Thin Layer Chromatography technique, preliminary surveys showed that Moroccan agricultural products including cereals appeared to be contaminated with spores of toxigenic strains of *Aspergillus*. Later, a series of analyses supported by the Direction of Frauds Repression (Ministry of Agriculture) between 1991 and 1992 showed that one corn sample was found to be contaminated with OTA [35].

Recently, we have carried out a study on the contamination of 60 samples of grains of cereals commercialized in Morocco with mycotoxins. Results showed that 40, 40 and 55% of analyzed samples of corn, wheat and barley were contaminated by OTA, respectively [36]. In barley samples, OTA levels varied between 0.04 and 0.8 ng/g, with an average concentration of 0.17 ng/g. In corn samples, the highest value found was 7.22 ng/g with an average value of 1.08 ng/g. In wheat samples, the OTA average level was 0.42 ng/g and the maximum level was 1.72 ng/g. 

Hajjaji *et al.* [37] investigated the co-occurrence of OTA and deoxynivalenol (DON) and the associated toxigenic fungi in 17 samples of wheat grain from Morocco. Authors reported that few samples were contaminated by the two mycotoxins (two samples for OTA and seven for DON). The main isolated fungi belong to the *Aspergillus*, *Penicillium and Fusarium* genera; only two strains of *A. alliaceus *and 14 strains of *A. niger* were able to synthesize OTA.

In Morocco, rice cultivation fluctuates vastly depending especially on climatic conditions. Of a potential area of 25,000 ha in the Gharb plain, the harvested area varies from 500 to 13,000 ha. On average the Moroccan population consumes 60,000 tons each year (2 kg/person/year). Due to the drought the country has endured over the last two decades, rice yield production decreased dramatically from 44,000 tons in 1993 to 2,500 tons in 1995, leading to extensive importation from other countries. Rice (Oryza sativa L.) is an important food crop worldwide, along with wheat and corn, and has been a major food in several countries. According to Park *et al.* [39], rice is naturally contaminated with *A. ochraceus* spores. Rice is an aquatic plant and is usually harvested at very high moisture levels (35-50%). Therefore, mycotoxin-producing molds could contaminate the grain and produce important quantities of OTA during storage. Furthermore, rice is a better substrate for the characterization of OTA producing *A. ochraceus* strains.

The first investigation on the presence of OTA in rice commercialized in Morocco reported that OTA contaminated 90% of total samples analyzed. Levels of contamination in positive samples ranged between 0.02 and 32.4 ng/g, where the average level of OTA in positive rice samples is 4.15 ng/g. 15% of total analyzed samples of rice exceeded the MRL of OTA set by the EU regulations [38]. In another study, Juan *et al*. [52] investigated the presence of OTA in 100 rice samples from five cities (Rabat, Témara, Salé, Casablanca and Méknès) in Morocco. Levels of OTA in positive samples ranged between 0.08 and 47 ng/g. The average contamination of all analyzed samples was 3.5 ng/g. The highest frequency of positive samples (30%) and the most contaminated sample (47 ng/g) was found in samples from Casablanca city, 14 out of 100 total samples exceeded the maximum level of 5 ng/g set by European regulations for OTA in cereals. Based in the results presented in this study, Juan *et al*. [52] estimated the daily intake of OTA in rice at 0.32 ng/kg b.w. for Moroccan consumers.

### 4.2. Breakfast and infants cereals

Breakfast cereals are generally made from principal ingredients destined for human consumption like wheat, rice, maize, barley and oat. Cereal grains are transformed to flakes and petals, and starch is cleaved to simple and digestible sugars. Theses cereals are often combined with honey, sugar, chocolate and dried fruits (raisins, bananas, nuts, *etc.*). Spoilage fungi are known to colonize most of these ingredients. The most important abiotic factors influencing the growth and OTA production by such spoilage fungi include water availability, temperature and when grain is moist, gas composition [40]. Breakfast cereals are generally commercialized in Morocco in small shops and supermarkets and consumed especially by children. Thus, more importance to their safety is needed. It should be clarified that most breakfast cereals available in Morocco are imported from foreign countries and little information is available about their quality during their entry into the country. The occurrence of OTA in 68 total analyzed samples of cereals products was studied using pressurized liquid extraction coupled to liquid chromatography method. Results showed that only four samples of breakfast cereals (two cornflakes samples (5.1 and 15.7 ng/g), one muesli sample (224.6 ng/g) and one fruits rings sample (127.5 ng/g)) were contaminated with OTA. Levels of OTA in positive samples ranged from 5.1 to 224.6 ng/g [41]. All positive samples (5.8% of total samples) were above the maximum level set by EU regulations for OTA in cereal products. However, all infant cereals analyzed in this survey were free of OTA contamination.

### 4.3. Bread

**Figure 2:**
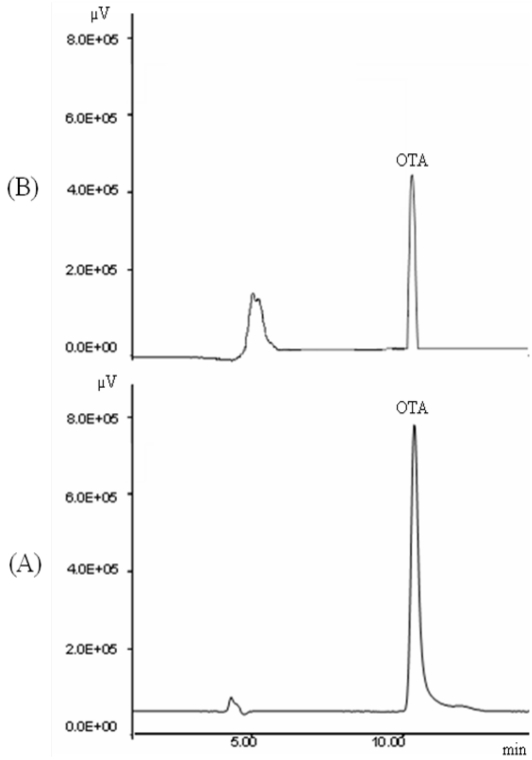
HPLC chromatograms of: (A) OTA standard solution (10 ng/mL); (B) a contaminated sample of bread containing 2.5 ng/g of OTA.

In Morocco, large amounts of cereals are consumed. Bread is the food most consumed by the population. Bread co is often homemade, especially in rural areas, but baker's yeast is frequently used rather than traditional sourdough starters. Nowadays, a change of food consumption habits has developed, with the increase in bread consumption, due to that the Moroccan's lifestyle has changed because of new working conditions. The presence of OTA in bread consumed in Morocco has been recently investigated [42]. A positive bread sample naturally contaminated with OTA is represented in Figure 2.

Results of this study showed OTA contamination of 48 out of 100 total analyzed samples. Levels of OTA in positive samples ranged between 0.14 and 149 ng/g, where the average level of OTA in positive samples was 13 ng/g. The highest frequency of positive samples (61.5%) and the most contaminated bread sample (149 ng/g) were found in the Casablanca area. In this survey, 26 % of total samples exceeded the maximum limit (3 ng/g) set for OTA in cereal products by EU legislation. 

Among cereal derived products, bread is of significant importance because it provides more nutrients to the population than any other single food and it is particularly important as a source of carbohydrates, proteins and vitamins. Bread is a product of daily consumption and highly demanded. The World Health Organization (WHO) recommends a 250 g/day intake, which corresponds to 90 kg/person/year. It was generally demonstrated that the main contributors to OTA intake are cereals and cereal products. Several authors have indicated bread as one of the main sources of daily intake of OTA. 

OTA daily intake was estimated from this study. Given that bread consumption in Morocco is estimated to 210 kg/year/person (*i.e.*,577 g/day/person), for an adult (60 kg b.w.), the estimated daily intake of OTA was calculated to be 126 ng/kg b.w./day. This value is seven times higher than the Tolerable Daily Intake (17.1 ng/kg b.w./day) set by the European Food Safety Authority [17], and nine times higher than the value set by the FAO/WHO Committee of Experts on Food Additives (14 ng/kg b.w./day) [16]. These results show that the Moroccan population is highly exposed to damaging effects of OTA and it can be speculated that the exposure could be related to cases of nephropathy widely reported in the country especially in young people of both sexes. However, this hypothesis needs to be confirmed especially by determination of OTA in biological fluids (blood, urine, breast milk *etc*.) in healthy individuals and from patients with renal dysfunctions (chronic renal insufficiency, chronic interstitial nephropathy, *etc*.) as has been performed in some North African countries like Tunisia, Algeria and Egypt [45].

### 4.4. Dried fruits and nuts

The Moroccan population consumes huge amounts of dried fruits directly or as ingredients included in special foods especially prepared during the ‘Ramadan’ fasting month and festival days. Almost all nuts such as pistachio, walnuts and peanuts consumed in Morocco are imported and little is known about their quality. Consequently, it is important to study the presence of mycotoxins, since there is a lack of information in the literature about their occurrence in these products. In Morocco, traditional techniques for the transformation and conservation of fruits are still used. These practices are very optimal conditions (especially temperature, humidity and fruits damages) for mold growth and mycotoxin production. The natural drying, which may consists of direct exposition of the fruit to the sun, is widely used, especially in rural areas. 

Fresh Fruits (raisins, figs *etc*.), having reached a sufficient degree of maturity, are gathered and transported to drying places such as the terrace of houses or a fenced piece of land to prevent the access of animals. These drying surfaces are generally exposed to a maximum of sun and are papered with herbs to avoid the contact with the ground. Fruits are spread over these surfaces without preliminary treatment. After drying, fruits are collected and stored. During the process of fruit drying, the sugar is concentrated as the moisture content decreases resulting in an almost selective medium for xerotolerant molds such as *A. niger* section nigri species. Among black aspergilli, *A. carbonarius* is the OTA producing isolate observed most frequently. Other black aspergilli including the *A. niger* aggregate and *A. aculeatus* have also been found to produce OTA on grapes. The incidence of AFs and OTA in dried fruits and nuts could be avoided or at least decreased if good agricultural and manufacturing practices from harvesting to processing were used. It should be mentioned that the project for mycotoxin regulations did not set limits for AFs and OTA in dried fruits and nuts. The presence of OTA in dried fruits and nuts from Morocco was studied by Zinedine *et al.* [38]. The authors reported that the incidence of OTA in dried raisins, dried figs, walnuts, and peanuts was 30%, 65%, 35%, and 25% respectively, while pistachio samples were free of OTA. The OTA average values in positive samples of peanut, dried figs, dried raisins and walnuts was 0.68, 0.33, 0.96 and 0.11 ng/g, respectively.

### 4.5. Beverages

Beverages are among the many food product groups at risk of contamination by harmful mycotoxins. These mycotoxins may form in an agricultural product before beverage manufacturing, or they may form during manufacturing. Beverages (wine, fruits juices and beer) produced in Morocco were analyzed by Filali *et al.* [43] for their content of OTA. The results from 30 wine samples; 20 red, seven white and three rosé, reported that OTA concentrations in the wines ranged from 0.028 to 3.24 µg/L with an overall median of 0.65 µg/L. The median concentration of OTA in white and rosé wines was found to be 0.117 µg/L, whereas that in red wines was 0.912 µg/L. The concentration of OTA in red wines ranged from 0.04 to 3.24 µg/L and those in the white and rosé wines from 0.028 to 0.540 µg/L. The red wines were thus more contaminated than white and rosé ones. The EU regulation set the acceptable limit for OTA in wine to 2 µg/L [40]. Thus, one sample containing 3.24 µg/L was above this limit. The results from analysis of 14 samples of various fruit juices (cocktail, orange, mango, peach, pineapple, clementine and grapefruit) show that only one sample (grapefruit juice) was contaminated, with a concentration of 1.16 µg/L. In analyzed beers, OTA was not detected. Almost all the grapes produced in Morocco are used for the wine industry. Grape juices are imported from Europe in very limited amounts and should not have a significant influence on the daily intake of OTA by the Moroccan population [43].

### 4.6. Olives

The production of olives in Morocco is about 6.9% of the global world production. The traditional harvest method used, and the long storage of fruits at ambient temperatures (18-28 °C) before processing, may result in a severe loss and a poor quality of olives. Micro-organisms involved in post-harvest alterations of the fruits before the fermentation processes were studied [46]. Many mold strains, in particular *Aspergillus* and/or *Penicillium*, are able to develop on olives and produce OTA and/or citrinin and/or AFs after harvest, during drying and storage of olives [47].

In Morocco, black table olives are prepared by an old process, which consists of drying and salting. The harvested black olives are filled in bags and salted (solid salt is sprinkled on the fruits while filling them in the bags). These bags are arranged one on the other and a heavy material (stone) is deposited on the top bag. The bitter black liquid is driven out under the action of weight and salt. A survey of the most frequent micro-organisms showed a low microbial load except for yeasts and molds. The most representative microbiota of black olives was species of mulds, which may be associated with food poisoning due to their mycotoxins [46]. On some occasions, phenomenally high concentrations of OTA have been reported in black olives, e.g., Maaroufi *et al.* [4] reported the contamination of one sample of black olives from Tunisia with a high level of 46,830 ng/g of OTA. 

The occurrence of toxigenic molds in black olives processed by the non-controlled traditional method is possible. Olives are among the commodities with high risk of mycotoxin contamination. Gourama and Bullerman [48] isolated toxigenic strains of *A. ochraceus* that produced ochratoxins from ‘Greek-style’ black olives produced in Morocco. A survey of the contamination of black olives commercialized in Morocco with mycotoxins reported that OTA was detected in 36% of total analyzed samples. OTA concentrations ranged from 0.62 to 4.8 ng/g with an overall median of 1.43 ng/g [49]. More recently, Roussos *et al.* [50] isolated strains of *A. flavus *and *A. niger*, from spoiled olive and olive cake of the 2003 and 2004 olive oil production campaigns in Morocco, that produced AFB1 and OTA. El Adlouni *et al.* [47] reported the presence of OTA, citrinin and AFs in black olive "Greek style" purchased from supermarkets and retail markets and concluded that the simultaneous presence of these toxins increases toxic risks and should spur authorities to control the conservation of olives especially after harvest.

## 5. Regulation of OTA

Mycotoxins are classified as the most important chronic dietary risk factor, higher than food additives, pesticide residues, plant toxins or synthetic contaminants. Since the 1960s, when the first AFB1 molecule was discovered, many countries have established maximum limits to protect health consumers against the risk of mycotoxins and to avoid the economic consequences of mycotoxin contamination. Various scientific and socio-economic factors play a role in the decision-making processes focused on setting limits for mycotoxins [23].

The regulation of mycotoxins in food and feed started in 1974, and since several countries have established or proposed maximum limits of mycotoxins in foods. By 1997, 77 countries had specific regulations for mycotoxins in different food and feed and 13 countries had general provisions, while about 50 countries did not have data available. The number of countries with specific regulations for mycotoxins has increased over the years. By the end of 2003, approximately 100 countries (covering approximately 85% of the world's inhabitants) had specific regulations or detailed guidelines for mycotoxins in food [51]. 

The regulations were related to traditional mycotoxins including OTA. According to the FAO document, 15 African countries were known to have specific mycotoxin regulations. These countries cover approximately 59 percent of the inhabitants of the continent. In Morocco, no mycotoxins regulations in food and feed are adopted by the authorities. However, a mycotoxin regulation project was prepared by the Joint ministerial committee for food control and frauds repression (CIPCARF). This project envisages the regulations of mineral and organic contaminants in food and feed and set maximum permissible limits of mycotoxins in certain food products intended for human and animal consumption. The proposed limit for cereals intended for human consumption is 30 ng/g for OTA. Even if, according to FAO documentation [51], Morocco has the most detailed mycotoxin regulations in comparison with some African countries, the proposed limit for OTA remains high and requires a revision. Also, limits for OTA in cereal products, beverages, and foods destined for children and babies *etc*. should be introduced before the final adoption of national regulation for mycotoxins in Morocco.

## 6. Conclusions and Perspectives

The occurrence of OTA in Moroccan foods as already investigated is presented in this paper. Levels of contamination were sometimes above the MRLs set by European regulations in food [53]. As reported, the Moroccan population could be exposed to risks of this toxin especially from cereals (wheat, corn, barley and rice), dried fruits and cereal products, *etc*. This situation should spur Moroccan authorities to devise prevention measures and set programs for surveillance of OTA in food. Nowadays, more than two million young people of both genders in Morocco are suffering from kidney problems (nephropathies) and the etiology of the disease is not well known; special attention should be given to the prevalence of OTA in foods most consumed in the country. Investigation of the implication of OTA in nephropathy cases is an urgency of public health by assessment of the exposure of patients and healthy people to OTA. 

Finally, because agricultural products constitute the most of exchanges between Morocco and its neighbors (EU countries), the Moroccan project for mycotoxin regulations needs to be harmonized with EU regulations especially for limits of OTA to protect both national and foreign consumers.
